# Supplementary material for: Incidence of snakebites in Can Tho Municipality, Mekong Delta, South Vietnam—Evaluation of the responsible snake species and treatment of snakebite envenoming
Source: PLoS Negl Trop Dis. 2020 Jun 17;14(6):e0008430. doi: 10.1371/journal.pntd.0008430 (PMC7323996; doi:10.1371/journal.pntd.0008430)
Supplement: Attachment — Submitted filename: Response to reviewers.docx [file pntd.0008430.s002.docx]

Letter with responses to reviewers‘ comments

We would like to thank the reviewers for their valuable comments to improve our manuscript entitled “Incidence of snakebites in Can Tho Municipality, Mekong Delta, South Vietnam – Evaluation of the responsible snake species and treatment of snakebite envenoming”.
Hereafter we address the reviewers’ comments provided on the different sections.

1. Methods

Reviewer #1: The aim of the study is clearly defined and is restricted to an area in South-Vietnam. The sample size is therefore limited, however, it is sufficient to answer the question to what extent snakebite causes health-problems. The statistical analysis has been correctly performed and there are no concerns about ethical standards.

Reviewer #2: The methods are well described and generally sound. The population under study is clearly defined and the sample size sufficient to reach conclusions on snakebites in the Can Tho municipality in Vietnam.  However, in the estimation of incidence based on community surveys, the authors excluded two districts because being mostly urban settings. But in the estimation of incidence based on hospital records, the authors used the total population of the Can Tho Municipality, hence including the two districts that were excluded in the other estimation. This creates a difference in the way incidence was estimated and therefore complicates the comparison between the two data on incidence. This issue is acknowledged by the authors when discussing the limitations of the study. Would it be possible to identify the locale where bites occurred from the hospital records and therefore to estimate the incidence from hospital records excluding the patients from the two central districts? This would allow the comparison between community-based data and hospital-based data.

Reviewer #3: The method of community survey may be helpful in situation where the community may not be in favour of seeking treatment at from a hospital. This is therefore appropriate for the locality of this study. This will certainly a good support/supplement to the formal hospital admission data. However, overlapping of the same patient may occur and should be taken into account.

Answer to Reviewer 2:

We identified two groups of snakebite patients according to their residence documented in hospital records in order to allow the comparison between community and hospital data. Out of 267 patients from Can Tho Municipality treated for snakebite in 2017, 140 patients were from the two urban districts Ninh Kieu and Binh Thuy, which were excluded from the community survey and 127 patients were from the seven other districts, which were included into the community survey.
Snakebite incidence was calculated separately at 36 and 14 snakebites per 100,000 persons per year in the two urban districts and in the 7 other districts, respectively.
Therefore the hospital records revealed approximately one third of snakebite incidence found in the community study in these 7 districts (14 versus 48).
However, the residence might not be the place where the snakebite happened, which was not indicated in the hospital records.
We included the changes in the methods (2.5.), result section (3.2.) and in the second section of the discussion.

Answer to Reviewer 3:
There were only a very small number of snakebite patients admitted to district hospitals and the majority of patients was transferred to the central levels to those two hospitals where antivenom was available. Most snakebite victims sought medical care directly in the central hospitals or they were sent directly to the central hospitals without being admitted and registered in the District hospital. However, we didn’t include patients from district hospitals in the evaluation of hospital records to avoid overlapping.
We explained this in the Method section (2.5.).

2. Results

Reviewer #1: There is only one table which summarizes the results of the study. The data evaluated are well presented and the results are clearly described.

Reviewer #2: In general, the presentation of results is clear. However, there are some limitations derived from the difficulties in getting precise information from hospital records: (1) The identification of the snakes responsible for the bites is always difficult when information is collected from hospital records. This is because the uncertainty in the identification of the snakes by patients or relatives. Since green snakes are relatively easy to recognize, this helps in the case of bites by Trimeresurus sp. The same occurs in the case of Naja sp bites. But the identification of non-venomous snakes is highly unreliable. I recommend only referring to non-venomous snakes without indicating the particular species or genera.

In line 220 and afterwards it is stated that “In both hospitals, snake antivenom against venom of Trimeresurus albolabris and Naja kaouthia from the Institute of Vaccines and Medical Biologicals (IVAC) in Nha
Trang, Khanh Hoa province, Vietnam was available. It has been administered to
405 (90%) out of 450 patients. All 5 patients after a cobra bite received monovalent
Naja kaouthia antivenom and 390 (94%) out of 414 patients with a green pit viper
bite received monovalent Trimeresurus albolabris antivenom”. This description of antivenoms used is a bit confusing. I gather from this description that there were two different monospecific antivenoms, one for cobras and one for green pit viper, but it remains unclear as whether there is a single bispecific antivenom. This needs to be better clarified in the text.

Likewise, the results indicating that no adverse reactions to antivenom administration are surprising as even the best antivenoms induce about 10-15% early adverse reactions. I suspect this is due to poor registration of adverse effects  in the hospital records. I suggest to the authors not including this information or discussing this possibility in the limitations of the study.

Reviewer #3: The result is as expected and answers the research questions as reflected in the objectives.

Answer to Reviewer 2:

During our community study there were 3 individuals bitten by green pit vipers and 4 by non-venomous snakes which they named as Copperhead rat snake, golden or paradise tree snake and Indochinese or oriental rat snake. We agree that correct identification of snakes by layman is unreliable and deleted the specific names (Results 3.1.).

In hospital files 414 cases of green pit viper bites, 5 cases of cobra bites were documented. In 31 cases the identification was not documented and there might be non-venomous snakebites in this group, however snake species were not identified.
The institute of Vaccines and Medical Biologicals in Nha Trang, Vietnam produces two different monovalent antivenoms, one against venom of Trimeresurus albolabris and one against Naja kaouthia. There is no bivalent antivenom available. We clarified this in the text (Results 3.2.).
We agree with reviewer 2 that poor registration is the reason for the fact that no adverse reactions to antivenom were documented in hospital records. We deleted the sentence “no side effects were recorded” (Results 3.2.).

3. Conclusion

Reviewer #1: All four criteria requested to be observed in the conclusion sections are met by the authors.

Reviewer #2: The conclusions are generally valid, especially regarding the relatively low incidence of snakebites in this setting. However, when explaining the low incidence of snakebites in this municipality, and comparing it to studies in Laos and Myanmar, the authors need to consider as another explanation the most prevalent snakes in these regions. It is likely that in regions where Daboia sp is prevalent, the likelihood of bites is higher than in regions where T. albolabris is prevalent. Thus, in addition to the explanations provided by the authors in terms of mechanization of agriculture, etc, the issue of predominant venomous snakes may also be considered.

Reviewer #3: There are a few limitations that needs to be addressed in order to obtain a more wholesome data. As suggested, the development of standardised guidelines and perhaps public awareness to seek formal treatment may increase the pick up rate of snakebites in this area. Obviously, increasing the competence level of healthcare professionals in treating snakebite envenoming will greatly improve the confidence in the public to seek the appropriate therapy. Improvement of the local healthcare system will greatly influence the overall outcome.

Answer to Reviewer 2:
We agree with reviewer 2 that the prevalent snake species in a certain region has an impact on incidence of snakebites. We addressed this point in the first section of the discussion.

Answer to Reviewer 3:
We agree that confidence of the public in the local healthcare system will change healthcare seeking behavior of snakebite victims. Only a whole package will achieve this and improve outcome of snakebite victims which includes (i) the sustained availability of effective snake antivenom, (ii) regular training to improve knowledge of healthcare personnel, (iii) information of community members and (iv) the development of guidelines for management of snakebite envenoming.

We added this in the last section of the discussion.

4. Editorial and data presentation modification

Reviewer #1: The authors mention in their community-based survey seven snakebite victims only, but much more in their survey  of snakebite patients from two hospitals. In the Discussion section, the seven victims are no longer mentioned. It should be discussed whether the community-based survey represents reliable data for estimating general snakebite incidence versus data of hospital patients. 
Line 285: the US-study of the effect of antivenom on limb functions. What means: "the study was underpowered"? Too few patients? Explain in a short sentence.

Reviewer #2: Minor revision.

Reviewer #3: Minor improvements.

Answer to Reviewer 1:

The community data represents reliable data, however the low number of 7 snakebite victims found in the community survey leads to a larger confidence interval of 20.5 – 99.8 in comparison with the evaluation of hospital data. We addressed this in the last section of the discussion. The direct comparison of snakebite incidence from community-based data and hospital records was possible after separating those patients from the hospital records, who lived in the seven districts which were included into the community survey (see also the answer to reviewer 2 in the section “Methods”).

The US-study was underpowered because the target sample size could not be reached. We explained this in a short sentence in the discussion section.
